# The silent storm: Quinine-induced Torsade de Pointes unmasking a latent KCNH2 variant

**DOI:** 10.21542/gcsp.2026.36

**Published:** 2026-08-31

**Authors:** Humberto Morais, Miguel Vicente, Catarina David, Fidel Manuel Caceres-Loriga

**Affiliations:** 1 Hospital Militar Principal/Instituto Superior; 2 Cardiovascular and Thoracic Centre–Clínica Girassol, Luanda, Angola; 3 Agostinho Neto University Faculty of Medicine, Luanda, Angola; 4 Doctors HealthCare, Florida, USA

## Abstract

Quinine, a *Cinchona* alkaloid and a cornerstone of malaria therapy in resource-limited settings, produces predominant sodium-channel blockade alongside modest inhibition of the rapid delayed-rectifier potassium current (IKr). Although generally safe at the population level, even modest IKr blockade can precipitate life-threatening Torsade de Pointes (TdP) in patients with severely impaired repolarization reserve. We report an 8-year-old boy with severe *Plasmodium falciparum* malaria who, on the third day of intravenous quinine, developed profound QTc prolongation (730 ms) followed by recurrent TdP. This arose from the convergence of quinine-induced IKr inhibition, hypokalaemia (K 2.9 mmol/L), and an underlying pathogenic KCNH2 variant, which together exhausted repolarization reserve. Although initially haemodynamically stable, the patient’s extreme electrical instability proved a sentinel event, prompting identification of latent Long QT Syndrome type 2 (LQT2). The case highlights the importance of ECG monitoring and electrolyte correction during quinine therapy. It illustrates how drug-induced arrhythmia can represent the phenotypic unmasking of diminished repolarization reserve, in which a pharmacological trigger exposes a silent genetic substrate—here necessitating lifelong beta-blocker therapy and family cascade screening to prevent sudden cardiac death.

## Introduction

Torsade de Pointes (TdP) is a life-threatening polymorphic ventricular tachycardia intrinsically linked to prolonged ventricular repolarization^1^. Although its hallmark—cyclic rotation of the QRS complexes around the isoelectric line—is pathognomonic, its management is distinct from that of other ventricular tachyarrhythmias, requiring rapid identification to prevent degeneration into ventricular fibrillation and sudden cardiac death^1,2^.

The QT interval reflects the duration of ventricular repolarization, governed by the balance between depolarizing (Na^+^/Ca^2^^+^) and repolarizing (K^+^) currents^3^. Within this framework, quinine occupies a singular historical and clinical position. Isolated in 1820, this *Cinchona* alkaloid remains a cornerstone of malaria therapy in sub-Saharan Africa, where limited access to artemisinin-based combination therapies (ACTs) often mandates its use as a first-line intervention^4^.

At the population level, quinine’s safety profile appears robust, but these aggregate figures can mask individual risk. Electrophysiologically, quinine differs from its diastereomer quinidine: whereas quinidine is a potent IKr blocker, quinine exerts predominant sodium-channel blockade with a comparatively modest effect on IKr. Nevertheless, in vulnerable individuals whose repolarization reserve is already diminished by electrolyte disturbance or latent genetic variants, even modest pharmacological IKr inhibition may trigger malignant ventricular arrhythmias^5–7^.

## Case Report

An 8-year-old boy with no prior cardiovascular history and no known family history of sudden cardiac death (SCD) presented to the emergency department with high-grade fever and persistent vomiting. Initial investigations confirmed severe malaria, with a thick blood smear showing a *P. falciparum* parasite density of 20 trophozoites per field. After failure of first-line chloroquine and with artesunate derivatives unavailable in the national health system at the time, intravenous quinine was initiated.

**Figure 1.**
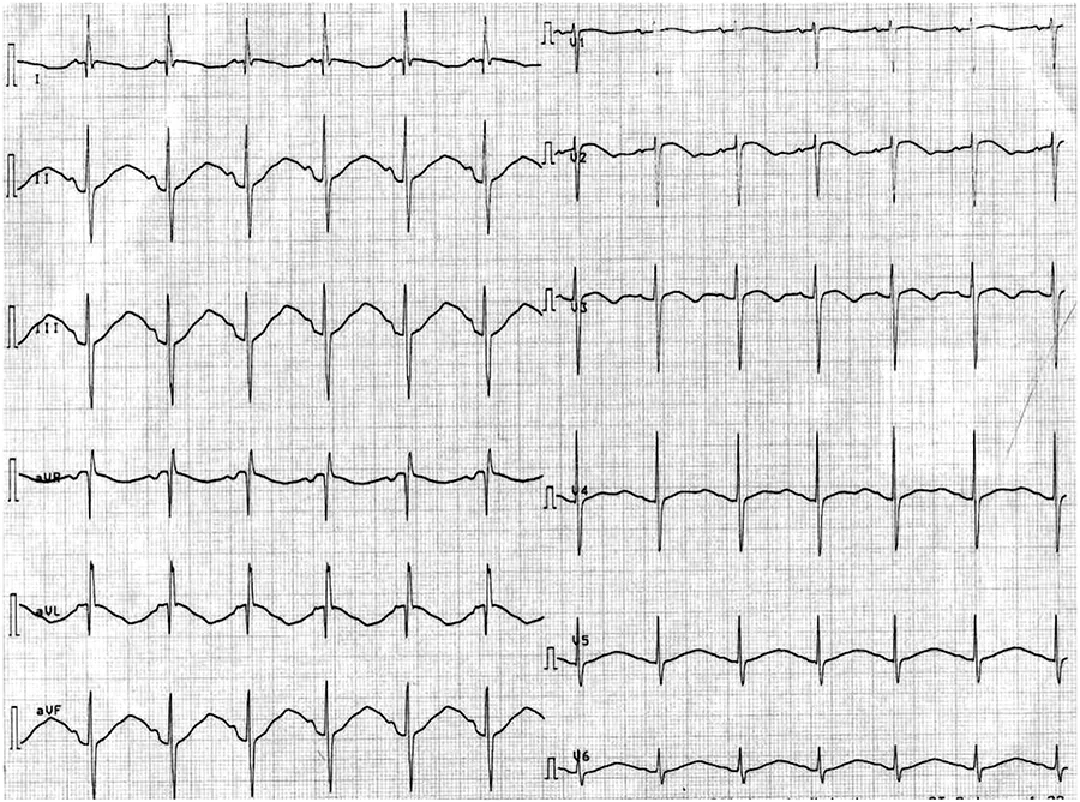
Admission 12-lead ECG showing marked QTc prolongation and notched T-waves in precordial leads (V2-V5), highly suggestive of LQT2.

**Figure 2.**
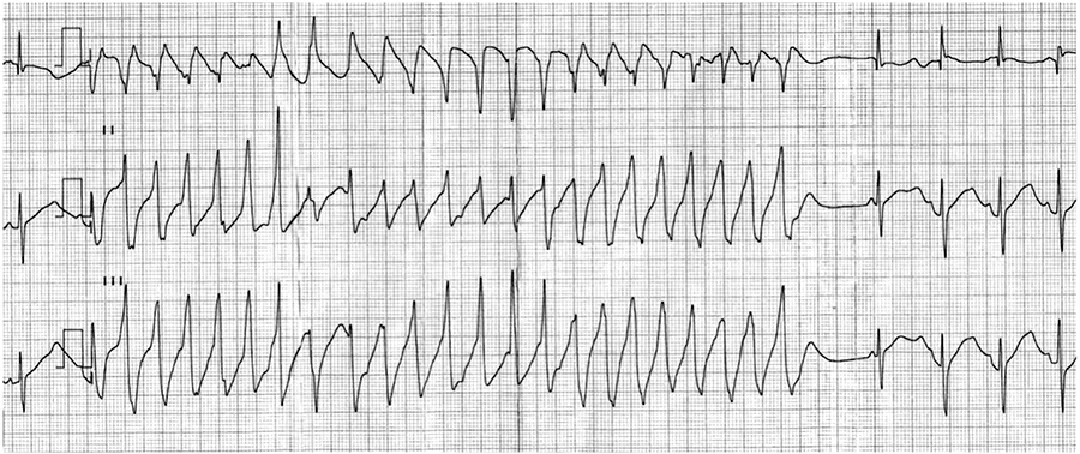
Rhythm strip capturing a paroxysm of *Torsade de Pointes* triggered by an “R-on-T” phenomenon.

**Figure 3.**
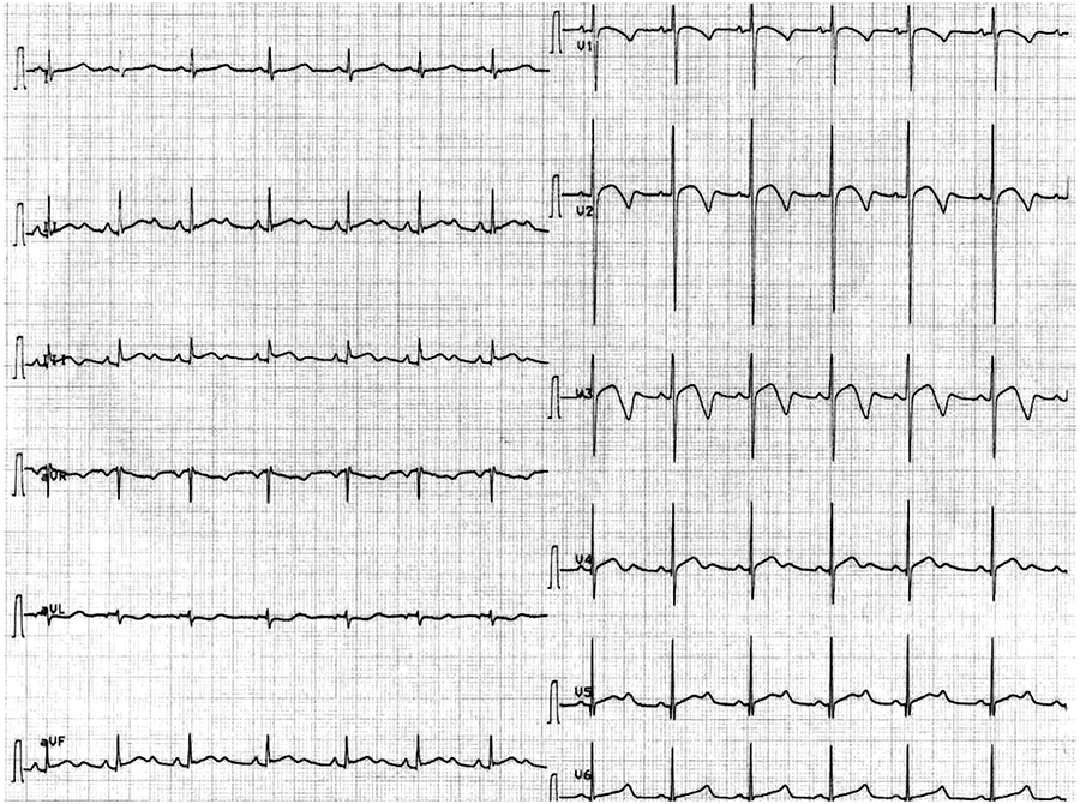
Follow-up ECG after magnesium sulfate and potassium replacement, showing significant regression of the QTc interval and electrical stabilization.

On the third day of treatment, while still haemodynamically stable, the patient suddenly developed retrosternal pain, palpitations, and near-syncope. An urgent ECG showed profound QTc prolongation of 730 ms (Figure 1), prompting immediate discontinuation of quinine and transfer to the Intensive Care Unit (ICU) for continuous monitoring. Despite cessation, he suffered multiple episodes of TdP (Figure 2). Laboratory analysis during the acute event revealed hypokalaemia (K^+^ 2.9 mmol/L), which aggravated the electrical instability. Acute management comprised a 1 g magnesium sulphate bolus followed by a weight-adjusted infusion, together with aggressive potassium replacement. Rhythm stabilised within 12 h, and the QTc subsequently fell to below 500 ms (Figure 3).

The patient made a favourable recovery and was discharged on the seventh day of hospitalisation, by which time his QTc had returned to below 500 ms. To ensure long-term safety, his mother was given an up-to-date list of QT-prolonging medications with strict instructions to avoid them. Genetic screening, facilitated by international collaboration, identified a pathogenic *KCNH2* variant, confirming congenital Long QT Syndrome type 2 (LQT2). Cascade screening subsequently revealed borderline QTc intervals in his father and sister, underscoring the value of family screening after a drug-induced arrhythmic event. At six-year follow-up, he remained free of recurrent symptoms on continuous non-selective beta-blocker therapy.

## Discussion

The clinical paradox of quinine lies at the intersection of its historical utility and its latent pro-arrhythmic potential. To our knowledge, this is the first documented case of quinine-induced TdP acting as a provocative “stress test” that unmasked a latent congenital LQT2 syndrome caused by a *KCNH2* variant.

Although quinine is a diastereomer of quinidine, at therapeutic concentrations it has a more modest effect on IKr and acts predominantly through sodium-channel blockade^8^. Our case, however, illustrates how a real-world convergence of insults can exhaust repolarization reserve entirely in a patient with extreme electrical vulnerability. This susceptibility was multifactorial. First, quinine directly inhibited IKr. Second, hypokalaemia (2.9 mmol/L) further reduced IKr conductance and increased susceptibility to drug-induced channel blockade. Third, the reverse use-dependence characteristic of IKr blockers is most pronounced at slower heart rates, further promoting QT prolongation and early afterdepolarizations^7,9^. This synergy accords with Jeyaraj et al.^10^, who showed that IKr blockade can specifically unmask abnormal repolarization in subjects with latent LQT2 variants. The underlying *KCNH2* variant provided the definitive substrate.

As shown by Ponce-Balbuena et al. ^11^, IKr is a heteromeric current formed by hERG 1a and 1b subunits, in which the Per–Arnt–Sim (PAS) domain of the 1a subunit is essential for generating the “tail current” that governs phase 3 repolarization. The observed phenotype is consistent with variants that reduce IKr amplitude through defective protein trafficking (Class 2). This finding carries additional weight because, under the ClinGen reclassification, *KCNH2* remains one of only seven genes with “definitive” evidence of causing LQTS^12^.

The absence of a family history of SCD is not unusual: incomplete penetrance often means an external trigger—here the “stress test” of quinine plus hypokalaemia—is required for the phenotype to manifest^13^.

## Limitations

Several limitations should be acknowledged. First, continuous ECG monitoring was not available during the initial 48 h of quinine therapy, so the earliest QTc changes could not be captured. Second, quinine plasma concentrations were not measured, which prevents us from excluding a supratherapeutic level as a contributing factor. Third, severe malaria—through its associated systemic inflammatory response and metabolic disturbance—may itself have acted as a pro-arrhythmic confounder. Finally, as a single case, our findings illustrate a specific mechanism of repolarization-reserve exhaustion rather than supporting any generalizable epidemiological conclusion.

## What have we learned?

• A drug-induced Torsade de Pointes event can be viewed as a ”pharmacological stress test”, capable of unmasking latent genetic substrates such as LQT2 that remain silent without an external trigger.
• Exhaustion of cardiac repolarization reserve is typically multifactorial. Here, the synergy between IKr blockade (quinine), metabolic disturbance (hypokalaemia), and an underlying *KCNH2* variant converged on a single repolarizing current, exceeding the heart’s electrical compensatory limits.
• The absence of a family history of sudden death does not exclude a hereditary channelopathy. Rigorous ECG monitoring and electrolyte management are therefore mandatory in patients receiving QT-prolonging medications, to expose hidden risk and prevent fatal outcomes.

## Author statement

Study design: HM and FMCL / Data collection: HM, MV Writing of the manuscript: HM, CD, MV / Revisions and approval of the final manuscript: All authors

## Conflicts of interest

The authors declare no conflicts of interest and no specific funding sources for this work.

## Consent

Written voluntary informed consent was obtained from the patient’s family. The study is in accordance with ethical principles.
